# Supplementary material for: Implementation of infection prevention and control practices in an upcoming COVID-19 hospital in India: An opportunity not missed
Source: PLoS One. 2022 May 23;17(5):e0268071. doi: 10.1371/journal.pone.0268071 (PMC9126379; doi:10.1371/journal.pone.0268071)
Supplement: Attachment — Submitted filename: my Comments.docx [file pone.0268071.s001.docx]

**Manuscript ID- PONE-D-22-00130**

The manuscript is well written, and it can be accepted after the minor revision. Please find my comments below.

1. Please provide some detailed information about COVID-19, it’s prevention strategies including role of health, immunity, and natural biomolecules i.e., doi.org/10.1007/s12088-020-00908-0; doi.org/10.1007/s12088-020-00893-4

2. Introduction, please include some quantitative information about cases, mortality, and casualty of COVID-19.

3. Discussion, please highlight minor information about COVID-19 variants and their challenges in its prevention.
