# Supplementary material for: Implementation of infection prevention and control practices in an upcoming COVID-19 hospital in India: An opportunity not missed
Source: PLoS One. 2022 May 23;17(5):e0268071. doi: 10.1371/journal.pone.0268071 (PMC9126379; doi:10.1371/journal.pone.0268071)
Supplement: Attachment — Submitted filename: Response to reviewers.docx [file pone.0268071.s002.docx]

| **Comments by the Reviewer 1** | **Authors’ responses to Reviewer’s comments** |
| --- | --- |
| 1) The only major issue with the manuscript is the presentation of the manuscript. There is no issue with the English of the manuscript. But the manuscript could be better organized. For example, it isn't very easy for the reader to differentiate between the content of Materials and Method section and Results section. | Complied.  The authors are grateful to the reviewer for his insightful remark which has helped us to organize the significant portions of the manuscript in order to make it easily understandable by the readers.  Changes are highlighted in lines 173-189 of the revised manuscript. |
| 2) Introduction: Minor information on the variants of COVID-19 and their future challenges can be included i.e. doi: 10.1007/s15010-021-01734-2. | Complied.  The authors want to thank the reviewer for the suggestion. The same issue has been raised by the other reviewers and addressed briefly under the discussion section.  Additions are highlighted in lines 383-391 of the revised manuscript. |
| 3) It would be required to provide one illustrative figure to summarize the whole study. | Complied.  A new illustrative Figure 5 in form of a flow diagram has been added and also cited in the revised manuscript (Lines 276-278). |
| 4) The authors should cross-check all abbreviations in the manuscript. Initially, define in full name followed by abbreviation. | Complied.  We have checked all abbreviations and as directed, all abbreviations have been expanded when they have mentioned first in the manuscript. |
| **Comments by the Reviewer 2** | **Authors’ responses to Reviewer’s comments** |
| •The manuscript is fairly excellent, but greater effort should be made to provide and debate current published studies on covid-19 waves and variations, as well as infection prevention and control techniques. | Complied.  Although the work of the manuscript was carried out during the period of the pandemic when variations in the virus was not known, we endeavored to mention the hardships which might be posed from infection control point of view due to the emerging variants in the subsequent waves under discussion section to comply with the reviewer’s suggestion. |
| • The last institutional meeting on IPC had taken place in 2018, November. However, under the guidance of the ICO, a few on-training clinical microbiologists from the Department of Microbiology had set an Infection Control Team (ICT). [If any reference available, please cite it] | As such, there is no reference.  This was an intra-institutional meeting. The copy of the circular of the mentioned meeting may be produced on case to case basis on request. |
| • In June 2019, initiatives for fresh sessions on infection control in form of lectures and demonstrations on hand hygiene, biomedical waste disposal, surface disinfection, needle stick injuries prevention and management, and introduction to antimicrobial stewardship program were initiated to update the healthcare personnel (HCP) based on the recently established national and international guidelines by the ICT. [If any reference available, please cite it] | The mentioned activity was conducted under the intra institutional teaching activities by the Infection Control Team.  The copy of the teaching schedule/ attendance records may be produced, if required. |
| • In table number 1 add references as another column. | Complied. The authors want to thank the reviewer for this important comment.  Accordingly in Table 1, a separate column has been added with references of different COVID-19 timeline events coinciding with the IPC training activities in the institute.  The additions are highlighted within the table. |
| • In the conclusion, add a few additional lines to better address the topic properly. In short, how your work is more valuable and novel than other reported studies till date. | Complied.  Following the important suggestion of the reviewer, we have re-written the conclusion.  Changes are highlighted in lines 394-400 of the revised manuscript |
| • Improve quality of figures. | Complied.  The quality of the figures has been substantially improved as per PLoS ONE requirements. |
| **Comments by the Reviewer 3** | **Authors’ responses to Reviewer’s comments** |
| 1. Please provide some detailed information about COVID-19, it’s prevention strategies including role of health, immunity, and natural biomolecules i.e., doi.org/10.1007/s12088-020-00908-0; doi.org/10.1007/s12088-020-00893-4 | The authors would like to appreciate the suggestion by the reviewer.  However, the authors feel that the present manuscript specifically focuses on the hospital infection prevention and control efforts made towards prevention and containment of COVID-19 disease in a dedicated COVID-19 treatment centre.  Although other strategies like immunization, biomolecules have substantial role in prevention, the same may not be relevant in context of the present manuscript. |
| 2. Introduction, please include some quantitative information about cases, mortality, and casualty of COVID-19. | Complied.  The authors want to thank the reviewer for this valuable comment for enriching the manuscript.  Additions are highlighted in lines 57-60 of the revised manuscript. |
| 3. Discussion, please highlight minor information about COVID-19 variants and their challenges in its prevention. | Complied.  The same has been addressed in response of the first comment of Reviewer 2.  Additions are highlighted in lines 383-391 of the revised manuscript. |
