# Supplementary material for: Implementation of infection prevention and control practices in an upcoming COVID-19 hospital in India: An opportunity not missed
Source: PLoS One. 2022 May 23;17(5):e0268071. doi: 10.1371/journal.pone.0268071 (PMC9126379; doi:10.1371/journal.pone.0268071)
Supplement: Attachment — Submitted filename: Response to reviewers.docx [file pone.0268071.s003.docx]

| **Comments by the Reviewer 1** | **Authors’ responses to Reviewer’s comments** |
| --- | --- |
| 1) The manuscript is well written. The Abstract, Introduction, and Material & Method are nicely written. However, the results section is not extensively reported in the manuscript. There are few key points discussed in the Discussion section that can be moved to the Result section. | Complied.  We are grateful to the reviewer for his kind words of appreciation. His further instructions for the revised manuscript have also been complied. We have also shifted some of the points from *Discussion* to *Results*.  Changes are highlighted in lines 254-257 and 260-263 of the revised manuscript. |
| 2) Please format the reference section according to Journal requirements. | Complied.  The authors want to thank the reviewer for the valuable suggestion.  The following changes have been made to the *Reference* section in the revised manuscript, as per the example given in the PLoS Instructions for authors.  i) The abbreviated names of the journals which were earlier italicized have been made non-italicized in the revised version.  ii) Month's name, which was mentioned after a year in few of the references, has been deleted from the references in the revised version.  iii) DOI i.e. Digital Object Identifier has been added in addition to traditional volume and page numbers of the cited references (as mentioned in the Submission Guidelines of PLOS ONE).  All changes are highlighted in yellow. |
